# Memory, Subjective Memory and Motor Functioning in Non-Demented Elders With and Without Parkinson’s Disease

**DOI:** 10.5964/ejop.v15i2.1672

**Published:** 2019-06-07

**Authors:** Maria Chiara Fastame, Paul Kenneth Hitchcott, Federica Corona, Giuseppina Pilloni, Micaela Porta, Massimiliano Pau, Maria Pietronilla Penna

**Affiliations:** a Department of Pedagogy, Psychology, Philosophy, University of Cagliari, Cagliari, Italy; b Department of Surgery, Medical, Molecular, and Critical Area Pathology, University of Pisa, Pisa, Italy; c Department of Mechanical, Chemical and Material Engineering, University of Cagliari, Cagliari, Italy; Webster University Geneva, Geneva, Switzerland; University of South Wales, Newport, United Kingdom

**Keywords:** Parkinson’s disease, subjective memory, objective memory, depression, gait, posture

## Abstract

Parkinson’s Disease (PD) is a progressive neurological condition characterized by motor and non-motor symptoms impacting life quality. The main aim of the current study was to investigate the effect of PD on objective (i.e., working memory and semantic memory) and subjective memory (i.e., self-reported seriousness of forgetting, mnemonic usage and actual memory efficiency) controlling for the effect of depressive symptomatology. The relationship of working memory performance to gait and mobility indices was also examined, as well as the factors predicting subjective memory were explored. Fifty-four community-dwelling adults (mean age = 72.3 years, SD = 8.8) were recruited in Sardinia, an Italian island located in the Mediterranean Sea. Specifically, 27 non-demented adults with mild, early-stage PD were matched for years of education, age, and gender with a sample of healthy individuals. Participants completed a test battery assessing objective memory, subjective memory, and depressive symptoms, and an instrumental analysis of gait and functional mobility was performed. Participants with PD had poorer objective memory across all indices measured and displayed a restricted set of gait and posture impairments. Working memory performance was selectively related to gait and posture measures. Moreover, participants with PD had lower trust in their memory efficiency relative to the past than the control healthy group. Finally, 22% of the variance in seriousness of the consequences of forgetting was predicted by education and general cognitive efficiency. Overall, the present findings confirm the presence of changes in both objective and subjective memory in PD, independent from depressive symptoms.

Parkinson’s Disease (PD) is a progressive degenerative condition defined by characteristic motor symptoms (e.g., bradykinesia, tremor, rigidity). However, non-motor symptoms, such as cognitive deficits (e.g., dementia, memory deficits, reduced impulse control) and behavioral disorders (e.g., depression, anxiety, apathy) are common even in the early stages of PD (Postuma et al., 2015; Rieu et al., 2016). Cognitive impairments typically involve visuo-spatial perception, working memory (e.g., active sequential information processing), executive functions (e.g., inhibition, planning, attentional set shifting) and declarative (e.g., episodic) memory (Di Rosa et al., 2017; Dubois & Pillon, 1996; see also Papagno & Trojano, 2018). During disease progression, cognitive (e.g., memory loss, word-retrieval difficulties) and behavioral (e.g., depression) symptoms become more tightly coupled as worsening balance and gait deficits increasingly restrict patients’ independence and quality of life (Barbosa et al., 2016; Bohnen & Cham, 2006; Dirnberger & Jahanshahi, 2013). Indeed, cognitive resources are necessary to process and integrate sensory and motor information and to plan the motor responses for maintaining balance (e.g., Kelly et al., 2015). There is evidence that working memory performance of individuals with PD is negatively impacted by the occurrence of depressive signs (Foster, Yung, Drago, Crucian, & Heilman, 2013) and that cognitive performance, primarily working memory and/or executive functioning, has been found to predict various behavioral outcomes in PD patients including risk of falling (Barbosa et al., 2016). Overall, the pervasive impact of PD symptoms is widely recognized to significantly impair life quality, especially in the advanced stages of the disease (e.g., Chrischilles, Rubenstein, Voelker, Wallace, & Rodnitzky, 1998; The Global Parkinson’s Disease Survey [GPDS] Steering Committee, 2001; Rieu et al., 2016).

While mnemonic processes such as working memory and declarative memory are crucial for many the day-to-day activities (e.g., naming objects, learning new words, remembering an appointment) measures of subjective memory are increasingly appreciated as important sources of information about informants’ risk of actual cognitive decline and awareness of cognitive functioning. Despite such recognition there have been relatively few studies of subjective memory and working memory in PD and no clear pattern of findings has emerged. This is significant since various aspects of subjective and objective memory are likely to play an important role in patient life quality, such as remembering to take medication, a crucial element in the daily life of a person with a chronic disease (Gould, McDonald-Miszezak, & King, 1997; Park & Mayhorn, 1996). A further reason why the investigation of subjective memory and mnestic efficiency in individual with PD is relevant is that memory complaints may reflect emerging functional brain abnormalities not easily detected via objective memory tasks. Furthermore, objective memory measures may identify individuals lacking awareness of mnestic impairment.

However, the existing literature is quite mixed even with respect to which aspects of subjective memory are impaired in PD (Ivory, Knight, Longmore, & Caradoc-Davies, 1999; Sitek et al., 2011; Souchay, Isingrini, & Gil, 2006). For instance, Sitek et al. (2011) observed accurate self-assessment of memory performance in PD both in relation to objective memory performance and the judgements of proxies. In contrast, Souchay et al. (2006) reported that while both healthy controls and people with PD underestimated their capacity to recall a set of words (i.e., global predictions of memory, when participants were asked to judge the likelihood of recall separately for each item, the group with PD overestimated memory accuracy. In addition, there are reports indicating that individuals with PD make less use of external (i.e., based on the use of environmental cues) memory strategies (e.g., diary, shopping list) than matched healthy controls, perhaps because such aids require greater fine motor efficiency (e.g., writing) (Johnson, Pollard, Vernon, Tomes, & Jog, 2005). Finally, the impact of PD on everyday cognitive performance may be selective rather than global. Poliakoff and Spark (2008) reported what while there was a non-significant increase in overall cognitive failures, significant increases on specific items related to distractibility and clumsiness were evident.

While increased clarity concerning the impact of PD on objective and subjective memory and the relationship between them is highly desirable, such a goal is complicated by confounding variance in depressive symptomatology (Dumas & Newhouse, 2015; Fastame, 2014). This is rarely considered in PD samples and, when it has, findings have been mixed (Coutler, 1989; Reynolds, Hanna, Neargarder, & Cronin-Golomb, 2017; Sitek et al., 2011). For instance, Sitek et al. (2011) reported an overestimation of memory defects in PD group due to self-reported depressive symptoms. In contrast, Coutler (1989) found no differences in metamemory monitoring between depressed and non-depressed patients with PD when compared with non-depressed healthy controls. Findings regarding the impact of depression on working memory are similarly mixed. Some studies have found depression to negatively impact performance (Dumas & Newhouse, 2015) while other studies have found no impairment (Reynolds et al., 2017). Overall, systematic studies investigating this topic are lacking and as suggested by Pannu and Kaszniak (2005), the administration of different neuropsychological tools to patients at different disease stages is also likely to contribute to the variability in findings.

The present study was undertaken with the aim of comparing subjective and objective memory function in non-demented people with and without PD independent from variance in depressive symptoms. The relationship of working memory to motor (gait and posture) performance was also examined. In short, the main aims of the current study were to examine:

1. the impact of PD at early stages of progression on objective memory processes, controlling for the effect of self-reported depression;
2. the impact of PD on subjective memory, controlling for the effect of self-reported depression and verbal long-term memory (i.e., vocabulary);
3. the impact of PD on gait and functional mobility;
4. whether disease status, general cognitive efficiency, self-reported depressive symptoms, education and hours per day spent reading predicted two indexes (i.e., Seriousness of Forgetting and Mnemonic Usage) of subjective memory;
5. the relationship between objective memory performance and quantitative measures of gait and functional mobility.

In relation to these aims the following hypotheses were tested:

1. Selectively poorer objective memory performance was expected in the PD group. On the digit span tasks, a deficit in backward but not forward immediate serial recall was predicted (Poliakoff & Spark, 2008). Moreover, no differences in terms of vocabulary were expected between the PD group and controls (Matison, Mayeux, Rosen, & Fahn, 1982; Wermuth, Knudsen, & Boldsen, 1996).
2. Self-reported depression was expected to impact (Dumas & Newhouse, 2015) or have no effect (Reynolds et al., 2017) on forward and backward immediate serial recall of non-demented adults with PD. No specific hypotheses can be proposed about the effect of depressive symptoms on vocabulary, because of the lack of previous evidence.
3. Poorer subjective memory was expected in the PD group. Across the dimensions of the Memory Functioning Questionnaire (MFQ, Gilewski, Zelinski, & Schaie, 1990), the PD groups was expected to have reduced strategy-use and worse memory efficiency relative to the past, and increased seriousness of forgetting (Ivory et al., 1999; Johnson et al., 2005). However, it could also be hypothesized that self-reported memory functioning was relatively preserved in participants with PD but impacted by depressive symptoms (Sitek et al., 2011).
4. Poorer gait and functional mobility was expected in the PD group (Corona et al., 2016; Palmerini, Mellone, Avanzolini, Valzania, & Chiari, 2013; Zampieri et al., 2010).
5. Significant correlations were hypothesized between working memory performance and postural measures (e.g., Dirnberger & Jahanshahi, 2013; Kelly et al., 2015).
6. To our knowledge no previous studies using the MFQ have been conducted in participants with PD. Nevertheless, subjective memory was expected to be predicted by general cognitive efficiency and self-reported depressive signs, since previous research has shown an association between memory complains and subtle cognitive decline and depression in preclinical patients with Alzheimer’s disease (Reisberg et al., 2008). Moreover, educational attainment was expected to predict the MFQ indexes, especially the use of mnemonic aids (Gilewski et al., 1990).

We believe this is the first study that has attempted to explore the nature of the relationship among measures of memory, MFQ subscales, depression and motor functioning in a sample of elders with and PD at the early stages. Such investigation in the early stages of the disorder may yield important knowledge about intervention strategies that may improve or preserve life quality.

## Method

### Participants

Fifty-four community-dwelling adults (mean age = 72.3 years, *SD* = 8.8) residing in the city of Cagliari took part. Twenty-seven participants with a PD diagnosis were matched for years of education, age, and gender with a group of peers without any signs of neurodegenerative or further chronic illnesses. Participants with PD were recruited via the Sardinian PD association (ASAMPA), the Neurology and Physical Rehabilitation Departments of the General Hospital “G. Brotzu” located in Cagliari. Non-PD controls were recruited at the University of the Third Age located in Quartu Sant’Elena, a village in the Cagliari hinterland. To be enrolled in the study, participants with PD had to satisfy the following inclusion criteria: diagnosis of PD carried out according to the UK Brain Bank criteria (Gibb & Lees, 1988), ability to walk independently and the absence of cognitive impairment, psychiatric or severe systemic pathological conditions. All participants were administered the MMSE as a screen for global cognition, however, the use of this instrument in PD has been questioned (Athey, Porter, & Walker, 2005; Zadikoff et al., 2008). For this reason, participants with an age- and education-adjusted MMSE score <24 and a subnormal Raven’s Progressive Matrices score (Italian validation, Belacchi, Scalisi, Cannoni, & Cornoldi, 2008; Raven, 1958) were excluded. These criteria resulted in the inclusion of two participants in the PD group with a borderline MMSE score. The part III of the Unified PD rating scale (UPDRS-III, Movement Disorder Society Task Force, 2003) was used to assess the level of motor impairment and disability (*M* = 18.5, *SD* = 10.2). The severity of the motor symptoms was assessed according to the modified Hoehn and Yahr (1967) scale (i.e., H&Y score) and the score ranged between 1 and 3 (*M* = 1.9, *SD* = 0.5). Control group participants had to show no sign of cognitive decline (≥24 MMSE score) and be free from any musculoskeletal and neurodegenerative diseases. Overall, all the participants were cognitively healthy (*M* = 26.4, *SD* = 1.8) and no differences were found between the group with PD and the control one in terms of MMSE score, *t*(52) = .19, *p* = .85.

Following similar previous research (e.g., Fastame & Penna, 2014; Hitchcott, Fastame, Langiu, & Penna, 2017), education was dichotomized in two levels: low (i.e., ≤8 years) and high (i.e., >8 years). Gender was counterbalanced across the groups, χ^2^(1) = .092, *p* = .76. Similarly, education level was counterbalanced in the PD and control groups, χ^2^(1) = 2.53, *p* = .11. This outcome was replicated when education was assessed in terms of total years of school attending, that is, no differences were found between the PD group and controls, *t*(52) = 1.53, *p* = .13. Overall the mean level of education of the current sample was relatively high (*M* = 13.9 years, *SD* = 5.2). Table 1 summarizes the socio-demographic and lifestyle main information of the participants.

**Table 1:** Socio-Demographic Information, Global Cognitive Efficiency Index (i.e., Mean MMSE Score) and Lifestyle Characteristics of the Participants With and Without PD Recruited for the Study

Characteristic	PD Group	Control Group
Sample size, *n*	27	27
Gender, *n*		
Males	20	19
Females	7	8
Marital status, *n*		
Single/widowed	5	6
Married/engaged	22	21
Living with, *n*		
Others	22	24
Alone	5	3
Education, *n*		
≤ 8 years	9	4
> 8 years	18	23
MMSE, *M* (*SD*)	26.41 (2.05)	26.5 (1.68)
Smoking, *n*		
Yes	2	3
No	25	24
Medicine intake, *n*		
Yes	27	21
No	0	6
Time spent reading, *n*		
Maximum 1 hour per day	19	18
Minimum 2 hours per day	8	9
Time spent watching television, *n*		
Maximum 1 hour per day	9	11
Minimum 2 hours per day	18	16
Outdoor hobbies, *n*		
Yes	23	24
No	4	3

*Note.* PD = Parkinson’s disease; MMSE = Mini Mental State Examination.

### Materials

Participants were presented the following inventories and tests:

1. The motor examination subscale of the Unified PD rating scale (UPDRS-III, Movement Disorder Society Task Force, 2003), that is composed of 14 items assessing PD-related motor impairment (i.e., rigidity, tremor and bradykinesia) and balance and mobility behaviors (i.e., postural stability, gait, posture and arising from a chair) on a four-point Likert scale (i.e., 0 indicates lack of impairment, whereas 4 indicates the severity of the impaired behaviors). As illustrated earlier, this tool provides an index of the disability level and motor symptoms.
2. The preliminary interview developed by Fastame and Penna (2012) to collect socio-demographic (e.g., gender, marital status) and lifestyle information about the participants.
3. The Mini-Mental State Examination (MMSE; Folstein, Folstein, & McHugh, 1975; Italian validation, Fava, 1983) that was presented to screen general cognitive efficiency. This test is composed of 20 items assessing spatial-temporal orientation, attention, short and long-term memory, visual–motor integration, and mental calculation abilities, respectively. The maximum total score is 30. In agreement with previous studies (Magni, Binetti, Bianchetti, Rozzini, & Trabucchi, 1996), scores were adjusted for age and years of education. As suggested by Turrina et al. (1993), a score ≤23 indicated the presence of suspected cognitive impairment.
4. The short Italian version of the Memory Functioning Questionnaire (Gilewski et al., 1990) validated by Pedone, Cosenza, and Nigro (2005) that was used to assess subjective memory. Specifically, 18 items of the Seriousness of Forgetting (i.e., it is an index of the estimated severity of the memory problems), eight items of Mnemonic Usage (i.e., that is an index of the esteemed use of external aids and memory strategies in the daily life) and three items of the Retrospective Functioning subscales were presented. For the retrospective functioning index, participants were asked to self-rate their actual memory functioning relative to 1, 5 and 10 years before. For each item the participant had to self-report the frequency of occurrence of each situation on a seven-point Likert scale ranging from 1 (i.e., always/very severe) to 7 (i.e., never/irrelevant). The sum of the scores relative to its items was computed for each subscale with possible total scores of 126 for Seriousness of Forgetting index and 56 for the Mnemonic Usage one. Retrospective functioning items were considered individually. People reporting higher scores have less trust in their memory processes, report less memory failures and use mnemonics (e.g., notes on a calendar) to recover crucial information less frequently.
5. The Centre for Epidemiological Studies of Depression Scale (CES-D; Radloff, 1977; Italian version, Fava, 1983) that provides a measure of perceived depressive signs. Each participant was asked to self-assess the occurrence of depressive symptoms during the past week on a four-point Likert scale (from 0, never or rarely to 3, most days or every day). The maximum total score is 60. According to the Italian normative data, a score ≥16 identifies risk of depressive illness, whereas a score ≥23 suggests the likely occurrence of clinical depression.
6. The Forward Digit Span Test (Wechsler, 1981; Italian validation De Beni, Borella, Carretti, Marigo, & Nava, 2007) that is a passive verbal working memory task assessing the efficiency of sequential processes. Each participant was asked to immediately recall an increasing sequence of digit numbers in the same order of presentation. The test is composed of 14 items each of which contains 3–9 digit numbers. Two sequences of the same length were presented until the participant failed in the immediate recovery of both of them, after that the administration of the task was ended. One score was given for each sequence correctly recalled. The maximum total score was 14.
7. The Backward Digit Span Test (Wechsler, 1981; Italian validation De Beni et al., 2007) is similar to the Forward Digit Span Test but has increased cognitive/attentional demand and was used to assess the efficiency of active sequential processes of verbal working memory. Specifically, each participant was asked to immediately recall the digit sequences presented by the experimenter in the reverse order. The administration and scoring procedures are identical to that described for the Forward Digit Span Test.
8. The Vocabulary Subtest of the Wechsler Adult Intelligence Scale (WAIS, Wechsler, 1981; Italian version, Orsini & Laicardi, 1997) that assesses the efficiency of semantic memory and is usually considered a measure of crystalized verbal intelligence. This task requires the definition (i.e., lexical retrieval) of 35 stimuli-target consisting of common words having high or low frequency of use in Italian. In agreement with the criteria provided in the manual of the test, scores assigned to each word definition range from 0 to 2, according to its correctness and completeness, that is, the highest score is given the more exhaustive definitions. The maximum total score is 70.
9. Three-dimensional computerized gait analysis, performed using a motion capture system composed of eight infrared cameras (Smart-D system, BTS Bioengineering, Italy) set at a frequency of 120 Hz. A number of anthropometric features (i.e., height, weight, anterior superior iliac spines distance, pelvis thickness, knee and ankle width, leg length) were collected, then 22 reflective passive markers (14 mm diameter) were placed on specific landmarks of individual’s lower limbs and trunk according to the protocol described by Davis, Õunpuu, Tyburski, and Gage (1991). Participants were asked to walk at a self-selected speed on a 10 m walkway for at least six times, allowing suitable rest where needed in order to avoid fatiguing effects. The acquired data were processed using dedicated software (Smart Analyzer, BTS Bioengineering, Italy) to calculate the following spatial-temporal parameters: gait speed, step length and width, cadence, stance, swing, and double support phase duration.
10. Instrumented Timed-Up-and-Go (TUG) test. This functional mobility test was carried out using a wearable inertial sensor (G-Sensor®, BTS Bioengineering S.p.A., Italy) previously employed for similar investigations in individuals of the same age range (Porta et al., 2018) that was attached to the subject's waist using a semi-elastic belt approximately at the L4-L5 inter-vertebral space position. To perform the test, participants were requested to sit on a standard office chair (seat height and width 48 cm, seat depth 40 cm) with a back support (34 cm high) and without armrests. Their arms were crossed at the wrists and held against the chest. Following a verbal start signal they stood up from the chair, walked for 3 m at a comfortable and safe speed, performed a 180° turn around a sign marked on the floor, walked back to the chair and performed a second 180° turn to sit down. The acceleration data measured along three orthogonal axes were acquired by the inertial sensor at 100 Hz frequency and transmitted via Bluetooth to a Personal Computer where dedicated software (BTS G-Studio, BTS Bioengineering S.p.A., Italy) was used to calculate the following set of parameters:TUG time (overall time needed to perform the test in s), sit-to-stand time (s), first (intermediate) 180° turning (time to perform the turn necessary to reverse the gait direction, s), second (final) 180° turning (time to perform the turn necessary to begin the sitting maneuver, s) and stand-to-sit time (time needed to sit down at the end of the task, s).

### Procedure

After having signed a written informed consent, participants were individually tested and interviewed in a quiet room at the Laboratory of Biomechanics and Industrial Ergonomics of the University of Cagliari, Italy over 2 consecutive days. During the first visit, gait and mobility were assessed; the psychological tools were administered on the second day. The psychological measures were presented in a partially randomized order. For all participants, general cognitive efficiency was assessed first then the socio-demographic interview was presented. After completing these instruments, the subjective memory and depression tools were presented; order of presentation was counterbalanced (Latin Square) across participants. The objective memory tests were always presented after the metamemory and depression questionnaires, in order to avoid any feed-forward effects following from memory self-assessment. To minimize fatigue among the oldest participants, for all participants an examiner read aloud the items contained in the questionnaire/test and recorded verbally reported responses. Each experimental session lasted about 60 minutes.

## Results

First, three separate Analyses of Covariance (ANCOVAs) were conducted to investigate the effect of group (i.e., participants with PD vs. healthy controls) on Forward Digit Span test, Backward Digit Span Test and vocabulary test conditions, in each case, controlling for the effect of self-reported depression. The effect of CES-D was not significant in any of the memory conditions, *F*(1,51) = 1.58, *p* = .21 for Forward Digit Span, *F*(1,51) = 2.78, *p* = .10 for Backward Digit Span and *F*(1,51) = 2.94, *p* = .09, respectively. The main effect of group was significant in terms of Forward, *F*(1,51) = 4.28, *p* = .04, $\eta_{p}^{2}$ = .08, and Backward, *F*(1,51) = 4.61, *p* = .04, $\eta_{p}^{2}$ = .08, Digit Span and Vocabulary, *F*(1,51) = 4.41, *p* = .04, $\eta_{p}^{2}$ = .08, efficiency. Controls outperformed participants with PD in Forward Digit Span (*M* = 6.72, *SD* = 1.5 vs. *M* = 5.69, *SD* = 1.9), Backward Digit Span (*M* = 6.03, *SD* = 1.4 vs. *M* = 5.04, *SD* = 1.8) and lexical competence (*M* = 53.30, *SD* = 15.2 vs. *M* = 43.82, *SD* = 17.1).

Next, to investigate the impact of PD on the two MFQ indexes while controlling for the effect of depression and semantic memory, a Multivariate Analysis of Covariance (MANCOVA) was carried out, using group (i.e., participants with PD vs. healthy controls) as between-subject variable and CES-D and vocabulary as covariates. The multivariate tests documented the significant main effects of CES-D (Wilks’ λ = .86, *df* = 2; 48, *p* = .03) and vocabulary (Wilks’ λ = .76, *df* = 2; 48, *p* = .009), whereas the main effect of group was not significant (Wilks’ λ = .96, *df* = 2; 48, *p* = .39). No group differences were found on Seriousness of Forgetting, *F*(1,49) = .67, *p* = .42) and Mnemonic Usage, *F*(1,49) = .91, *p* = .34, sub-scales. Participants with PD had similar levels of complaints about the seriousness of their memory failures (*M* = 76.43, *SD* = 3.7, than matched controls (*M* = 71.90, *SD* = 3.8). Moreover, the clinical group reported a similar use of external and internal memory strategies (*M* = 29.6, *SD* = 2.1) compared to the healthy controls (*M* = 32.6, *SD* = 2.2). The main effect of CES-D was significant in Seriousness of Forgetting condition, *F*(1,50) = 7.66, *p* = .008, $\eta_{p}^{2}$ = .13, but not in Mnemonic Usage one, *F*(1,50) = .20, *p* = .66, sub-scale. A similar pattern of results was found for vocabulary, *F*(1,50) = 14.69, *p* < .0005, $\eta_{p}^{2}$ = .23 for Seriousness of Forgetting and *F*(1,50) = .0001, *p* = .99 for Mnemonic Usage.

Furthermore, three separate ANCOVAs where conducted to explore the impact of group on self-assessed actual memory efficiency relative to 1, 5 and 10 years before, using both CES-D and vocabulary as covariates. For self-assessed memory, relative to 1 year earlier, there were main effects of group, *F*(1,50) = 8.73, *p* = .005, $\eta_{p}^{2}$ = .15, and CES-D, *F*(1,50) = 11.83, *p* = .001, $\eta_{p}^{2}$ = .19, whereas the effect of vocabulary was not significant, *F*(1,50) = .47, *p* = .49. The same pattern was found for self-assessed memory relative to 5 years earlier, *F*(1,50) = 12.69, *p* = .001, $\eta_{p}^{2}$ = .20 for group; *F*(1,50) = 10.63, *p* = .002, $\eta_{p}^{2}$ = .17 for CES-D and *F*(1,50) = 2.72, *p* = .11 for vocabulary) and to 10 years earlier, *F*(1,50) = 5.40, *p* = .02, $\eta_{p}^{2}$ = .10 for group; *F*(1,50) = 4.70, *p* = .03, $\eta_{p}^{2}$ = .09 for CES-D and *F*(1,50) = .03, *p* = .86 for vocabulary. Overall, participants with PD reported less trust in their memory functioning compared to the past. Moreover, people with PD reported more depressive signs (*M* = 16.44, *SD* = 9.31) than controls (*M* = 11.37, *SD* = 8.5). Table 2 summarizes the findings relative to all the above mentioned ANCOVAs and MANCOVA.

**Table 2:** Summary of ANCOVAs and MANCOVA Analyses Examining the Effect of Group (Participants With PD vs. Controls) on the Memory Measures (i.e., Forward Digit Span, Backward Digit Span and Vocabulary Tests) and Metamemory Dimensions (i.e., Seriousness of Forgetting, Mnemonic Usage and Retrospective Memory)

Dependent Variable	*df*	SS	MS	*F*	*p*	$\eta_{p}^{2}$	*M*_PD_	*M*_controls_
Forward Digit Span							5.69	6.71
Main effect Group	1	13.10	13.10	1.58	.04	.08		
Covariate CES-D	1	4.85	4.85	1.58	.21			
Backward Digit Span							5.10	5.90
Main effect Group	1	12.28	12.28	4.61	.03	.08		
Covariate CES-D	1	7.42	7.42	2.79	.10			
Vocabulary							43.82	53.30
Main effect Group	1	1117.21	1117.21	4.41	.04	.08		
Covariate CES-D	1	744.99	744.99	2.94	.09			
MFQ- Seriousness of Forgetting							76.43	71.90
Main effect Group	1	233.23	233.23	.67	.42			
Covariates CES-D	1	2678.31	2678.31	7.66	.008			
Vocabulary	1	.001	.001	14.69	< .001			
MFQ- Mnemonic Usage							29.60	32.63
Main effect Group	1	104.23	104.23	.91	.34			
Covariates CES-D	1	22.48	22.48	.20	.66			
Vocabulary	1	.001	.001	< .001	.99			
MFQ- Retrospective functioning compared to 1 year before							3.66	4.53
Main effect Group	1	8.60	8.60	8.73	.005	.15		
Covariates CES-D	1	11.66	11.66	11.83	.001	.19		
Vocabulary	1	.46	.46	.47	.49			
MFQ- Retrospective functioning compared to 5 years before							3.24	4.46
Main effect Group	1	17.10	17.10	12.69	.001	.20		
Covariates CES-D	1	14.33	14.33	10.63	.002	.17		
Vocabulary	1	3.67	3.67	2.72	.10			
MFQ- Retrospective functioning compared to 10 years before							2.89	3.88
Main effect Group	1	11.29	11.29	5.40	.02	.10		
Covariates CES-D	1	9.84	9.84	4.70	.03	.09		
Vocabulary	1	.06	.06	.03	.86			

*Note.* ANCOVA = analysis of covariance; MANCOVA = multivariate analysis of covariance; CES-D = Centre for Epidemiological Studies of Depression Scale; MFQ = Memory Functioning Questionnaire; PD = Parkinson’s disease; SS = Sum of Squares; MS = Mean Square. Self-reported depression (i.e., CES-D) with and without vocabulary score was used as covariate respectively for the MANCOVA and ANCOVAs.

Then, two separate stepwise regression analyses were conducted to explore whether group (i.e., people with PD vs. controls), general cognitive efficiency (i.e., MMSE), depression (i.e., CES-D), years of education, hours per day spent for reading (i.e., ≤1 hour vs. ≥2 hours) predicted Seriousness of Forgetting and Mnemonic Usage MFQ indexes. When Seriousness of Forgetting was entered as the dependent variable, it was found that years of education (*b* = -0.43, *t* = -3.53, *p* = .001) and MMSE score (*b* = 0.29, *t* = 2.35, *p* = .02) predicted 22% of the variance in the abovementioned MFQ subscale, corrected *R*^2^ = 0.22, *F*(1,50) = 8.38, *p* = .006. In contrast, none of the independent variables predicted the Mnemonic Usage measure.

To compare the impact of disease status on gait and functional mobility measures a series of *t*-test comparisons were conducted. The effect of group (control vs. PD) was significant for the step length, *t*(46) = -2.27, *p* = .03, and approached the significance for the time of the first 180° (intermediate) rotation in TUG, *t*(46) = 1.81, *p* = .08. The mean step length of participants with PD was shorter (*M* = .57, *SD* = 0.10) than that of matched controls (*M* = .67, *SD* = 0.21).

Finally, a series of Pearson’s product moment coefficients were computed to investigate the relationship between Forward and Backward Digit spans and the postural motor indexes. Statistically significant correlations were found between Forward Digit span and step length (*r* = .37, *p* = .009), TUG sit-to-stand time (*r* = -.30, *p* = .049), TUG stand-to-sit time (*r* = -.39, *p* = .010) and time of final 180° turning in TUG (*r* = -.37, *p* = .015), respectively. In contrast, Backward Digit span significantly correlated only with step length (*r* = .30, *p* = .037) and approached significance with overall TUG time (*r* = -.29, *p* = .057). Table 3 illustrates these outcomes.

**Table 3:** Pearson's Product-Moment Correlation Matric Among Working Memory Measures (i.e., Forward Digit Span and Backward Digit Span) and Postural Motor Indexes (i.e., Cadence-Step-min, Stride-Length-M, Step-Length-M, Step-Width-M, TUG-Time, TUG-sit-to-stand-time, TUG-stand-to-sit-time, TUG-first-180°-turning, TUG-second-180°-turning)

Variable	1	2	3	4	5	6	7	8	9	10	11
1. Forward Digit Span	–										
2. Backward Digit Span	.51**	–									
3. Cadence-Step-min	-.01	.05	–								
4. Stride-Length-M	.27	.17	-.69**	–							
5. Step-Length-M	.37**	.30*	-.51**	.94**	–						
6. Step-Width-M	.09	.03	-.77**	.95**	.80**	–					
7. TUG-time	-.27	-.29	.008	-.57**	-.67**	-.45**	–				
8. TUG-sit-to-stand-time	-.30*	-.12	-.22	.10	-.004	.18	.18	–			
9. TUG-stand-to-sit-time	-.39*	-.21	-.41**	-.57**	-.54**	-.002	.55**	.49**	–		
10. TUG-first-180°-turning	-.19	-.20	-.23	-.32*	-.29	.18	.35*	.51**	.59**	–	
11. TUG-second-180°-turning	-.37*	-.25	-.19	-.46**	-.40**	.21	.39*	.44**	.65**	.72**	–

*Note.* TUG = Timed-Up-and-Go.

*p < .05. **p < .01.

## Discussion

Only a few studies have examined the subjective memory deficits that can accompany objective memory impairment in the early stages of PD and these have yielded inconsistent findings. The present study aimed to resolve this issue via concomitant measurement of depressive symptomatology and re-examine the association between objective memory and motor function. The sample was comprised of community dwelling individuals, with or without PD, all of whom were screened for signs of global cognitive impairment. The control and PD groups were age-, education- and sex-matched yet compared to the control group displayed objective memory impairment across all indices examined; forward and backward digit spans were lower, as was performance on the vocabulary subtest of the WAIS. These tests assess distinct components of memory (passive and active working memory and semantic memory, respectively) commonly reported to be impaired in PD (Di Rosa et al., 2017; Gabrieli, Singh, Stebbins, & Goetz, 1996; Henry & Crawford, 2004). The deficit in both forward and backward digit span was somewhat unexpected since demand characteristics can influence task sensitivity (Poliakoff & Spark, 2008). Indeed, the lowest accuracy of mnestic functions of people with PD was detected when an active manipulation of verbal serial information involving executive functions was requested (i.e., Backward Digit Span Test condition), and even when a reduced amount of cognitive resources was used to retrieve the sequential strings in forward order. Similarly, despite similar education and global cognitive efficiency levels characterizing participants with and without PD, unexpectedly, differences in terms of vocabulary were found between the two groups (e.g., Matison et al., 1982; Wermuth et al., 1996). For what concerns the vocabulary performance, a possible explanation is that our participants with PD were older that those recruited by Wermuth et al. (1996) and, in turn, the aging factor could be related to the lowest efficiency of the attentional/control processes of the participants with PD that was reflected in poorer working memory functions. However, at present we cannot exclude that the lowest vocabulary efficiency of the group with PD could be the expression of a more general impairment of semantic memory (Henry & Crawford, 2004; see also Gabrieli et al., 1996) or of a specific word-finding difficulty (e.g., Matison et al., 1982) that have been observed in that clinical population. Future research has to clarify this issue by the administration of specific tasks assessing this (e.g., semantic fluency tasks).

Use of the MFQ permitted assessment of several aspects of subjective memory and revealed contrasting consequences of PD. Control and PD groups rated the seriousness of forgetting similarly and, unlike some previous research (Johnson et al., 2005), use of memory aids did not differ. These are odd outcomes given the evident impairment of objective memory and wider evidence showing that non-demented PD patients often experience difficulties completing everyday tasks (Poliakoff & Spark, 2008). Two possible interpretations can be suggested. First, the PD patients may have been unaware of their declarative and working memory deficits and, as a result, did not compensate with increased use of memory aids. However, this seems unlikely, since self-rated memory performance (relative to 1, 5 or 10 years previous) was worse in the PD group suggesting intact awareness of cognitive decline. A second, and more likely, scenario is that motivation to use memory aids was reduced. Apathy is a recognised feature of PD, known to be associated with cognitive functioning and independent from disease progression (Pluck & Brown, 2002). This would appear to provide a better explanation for the observed pattern of differences in subjective memory. Despite from these suggestions, from an applied viewpoint, the current outcomes could have serious consequences in terms of medication adherence (Gould et al., 1997) and suggest that our participants with PD could benefit of memory and metamemory interventions promoting life quality and enhancing the ability to deal with everyday problems.

One implication of these findings is that concomitant targeting of reduced motivation may be a more effective means of developing self-regulation of memory impairments in PD. Such an approach could be especially useful in those with depression (Goedeken, Potempa, Prager, & Foster, 2018) given that our findings confirmed that increased depressive symptomatology was associated with impairments of both objective and subjective memory (e.g., Dumas & Newhouse, 2015; Fastame, 2014). However, additional research in this area is needed since prior research indicates that apathy and depression are not correlated in PD (Pluck & Brown, 2002).

Moreover, education and general cognitive efficiency predict seriousness of forgetting, but not memory usage and self-reported depressive signs did not predict any MFQ measure. Overall, these outcomes are partially consistent with previous evidence (Gilewski et al., 1990; Reisberg et al., 2008) suggesting a need for further investigation.

Motor function was impaired in participants with PD, as expected; they walked with shorter steps and performed the 180° turn during the TUG task less effectively. Most previous studies on gait and mobility have reported similar gait and TUG anomalies in individuals with PD (Boonstra, van der Kooij, Munneke, & Bloem, 2008; Morris, Morris, & Iansek, 2001). That only a restricted set of motor impairments was evident is consistent with the mild disability level of the PD group (as indicated by their H&Y scale scores) and the fact that they were tested while on medication. The observed significant correlations between digit span performance and indices of gait and posture are of greater interest. Although previous studies have made similar observations (e.g., Kelly et al., 2015), the present study confirmed that these associations are independent of variance in depressive symptoms. Previous research shows that depression is frequently present in PD and that it may confound both motor and cognitive task performance (Cummings, 1992).

The most obvious limitations of the present research concern our sample and its size and composition. Participants were non-demented, community-dwelling adults with or without early-stage PD; those with PD were medicated. Overall, the sample had a relatively high education level and was actively engaged in leisure activities. Extending the findings beyond this small homogenous group requires further, larger scale investigation. A further point is that the sample was recruited in Sardinia, a region in which the prevalence of PD is reduced compared to the mainland Italy and Northern Europe (Pupillo et al., 2016; Rosati et al., 1980). Perhaps of greater significance is that older adults from this region have consistently been found to display unusual psychological characteristics relative to age-matched peers. These include some attributes examined in the present study (e.g., Fastame, Hitchcott, & Penna, 2015; Fastame & Penna, 2014; Hitchcott et al., 2017; Hitchcott, Fastame, & Penna, 2018) and the extent to which this influenced the present findings is unclear without further investigation. In conclusion, future research extending these findings to broader groups with PD and investigation of the neural substrates underpinning the metamemory dimensions explored in the current study would be useful.
